# Intracranial recordings show evidence of numerosity tuning in human parietal cortex

**DOI:** 10.1371/journal.pone.0272087

**Published:** 2022-08-03

**Authors:** Jelle A. van Dijk, Maartje C. de Jong, Gio Piantoni, Alessio Fracasso, Mariska J. Vansteensel, Iris. I. A. Groen, Natalia Petridou, Serge O. Dumoulin

**Affiliations:** 1 Spinoza Centre for Neuroimaging, Amsterdam, The Netherlands; 2 Experimental Psychology, Utrecht University, Utrecht, The Netherlands; 3 Department of Psychology, University of Amsterdam, Amsterdam, The Netherlands; 4 Amsterdam Brain and Cognition (ABC), University of Amsterdam, Amsterdam, The Netherlands; 5 Radiology Department, Imaging Division, Center for Image Sciences, University Medical Center Utrecht, The Netherlands; 6 Institute of Neuroscience and Psychology, University of Glasgow, Glasgow, United Kingdom; 7 UMC Utrecht Brain Center, Department Neurology and Neurosurgery, UMC Utrecht, Utrecht, The Netherlands; 8 Informatics Institute, University of Amsterdam, Amsterdam, The Netherlands; 9 Department of Psychology, New York University, New York, United States of America; 10 Experimental and Applied Psychology, VU University, Amsterdam, The Netherlands; Harvard Medical School, UNITED STATES

## Abstract

Numerosity is the set size of a group of items. Numerosity perception is a trait shared across numerous species. Numerosity-selective neural populations are thought to underlie numerosity perception. These neurons have been identified primarily using electrical recordings in animal models and blood oxygen level dependent (BOLD) functional magnetic resonance imaging (fMRI) in humans. Here we use electrical intracranial recordings to investigate numerosity tuning in humans, focusing on high-frequency transient activations. These recordings combine a high spatial and temporal resolution and can bridge the gap between animal models and human recordings. In line with previous studies, we find numerosity-tuned responses at parietal sites in two out of three participants. Neuronal populations at these locations did not respond to other visual stimuli, i.e. faces, houses, and letters, in contrast to several occipital sites. Our findings further corroborate the specificity of numerosity tuning of in parietal cortex, and further link fMRI results and electrophysiological recordings.

## Introduction

Perception of numerosity, i.e. the set size of a group of items, is implicated in many cognitive functions [1] and processes such as multiple object tracking [2]), mathematics [3–5], decision-making [6], and dividing attention [7]. Behavioural studies have shown that numerosity perception is a trait shared across a wide range of species, e.g. non-human primates [8–10], non-primate mammals [11], birds [12]and fish [13]. Furthermore, numerosity perception is also found in different stages of development, e.g. human infants [3] and children [14]. Neuronal populations selective for numerosity have been found using electrophysiological recording in corvids [15,16], monkeys [6,17–20], and using depth electrodes in humans [21].

Studies utilizing functional magnetic resonance imaging (fMRI) have revealed a network of cortical and subcortical regions that are involved in numerosity processing [7,14,22–26]. Recently, we have revealed that a number of locations involved in numerosity processing are topographically organized, with numerosity preference gradually changing along the cortical surface [27–30], a discovery made feasible by ultra-high field strength MRI at 7 Tesla [31]. Neuronal populations in these topographic regions show numerosity tuning, with large responses for stimuli of a specific numerosity and increasingly smaller responses when the numerosity of a stimulus is further from the preferred numerosity of that population. This is in line with electrophysiological recordings for numerosity in animals, where spiking rates of (a group of) numerosity-selective neurons decrease with distance from the preferred numerosity. Both animal electrophysiology and human fMRI recordings of numerosity tuning implicate the involvement of the parietal lobes. However, the only human electrophysiological recordings demonstrating numerosity were made in medial temporal lobe [21]. Involvement of parietal and temporal regions are not mutually exclusive given the large network thought to be involved in numerosity processing [7,14,22–26,28].

Here, we used intracranial recordings from the parietal and occipital lobe with subdural electrode grids or stereo-electroencephalography (S-EEG) needles to measure responses to different visual numerosities. Intracranial recordings provide a great temporal (millisecond (ms) range) and spatial resolution (millimetre (mm) range). High spatial resolution is needed as the extent of cortical numerosity maps -the parietal numerosity maps in particular (Numerosity maps in Parietal Cortex (NPC)-complex)- encompass roughly 500 mm^2^ of flattened cortical surface, and these maps are further obscured by cortical folding [28]. To facilitate the comparison between intracranial recordings with fMRI findings, we used stimuli derived from those used in previous fMRI studies that have found numerosity-tuned cortical areas [28,29]. We found numerosity-selective response patterns in locations corresponding with the most robustly identifiable numerosity-selective topographic map in fMRI studies (NPC maps; [28–31]. We provide several control analyses showing that the response preferences of these electrodes are not in line with category specific visual stimuli, further supporting the numerosity-selective nature of neural population responses in this cortical location.

## Methods

### Participant information

Participants were three individuals with drug-resistant epilepsy who were candidate for resective surgery. They were admitted for intracranial epilepsy monitoring at the University Medical Center Utrecht, and implanted with subdural ECoG grids or depth electrodes as diagnostic procedure for surgical treatment. The study was approved by the ethical committee (METC) of the University Medical Center Utrecht, and was conducted in accordance with the Declaration of Helsinki (2013). All participants gave written informed consent to participate in this research.

Electrodes were implanted for a week as part of epilepsy treatment, for localization of the epileptic focus. Grid electrodes (AdTech, Racine, WI) had a measurement surface of 2.3 mm diameter, with 1 cm inter-electrode spacing, and were positioned directly on the cortical surface. A reference electrode was positioned extra-cranially on the mastoid bone. S-EEG needle electrodes (DIXI Medical) had a diameter of 0.8 mm, interelectrode distance (’Insulating Spacer Length’) of 1.5 mm and contact length of 2 mm.

**Fig 1:**
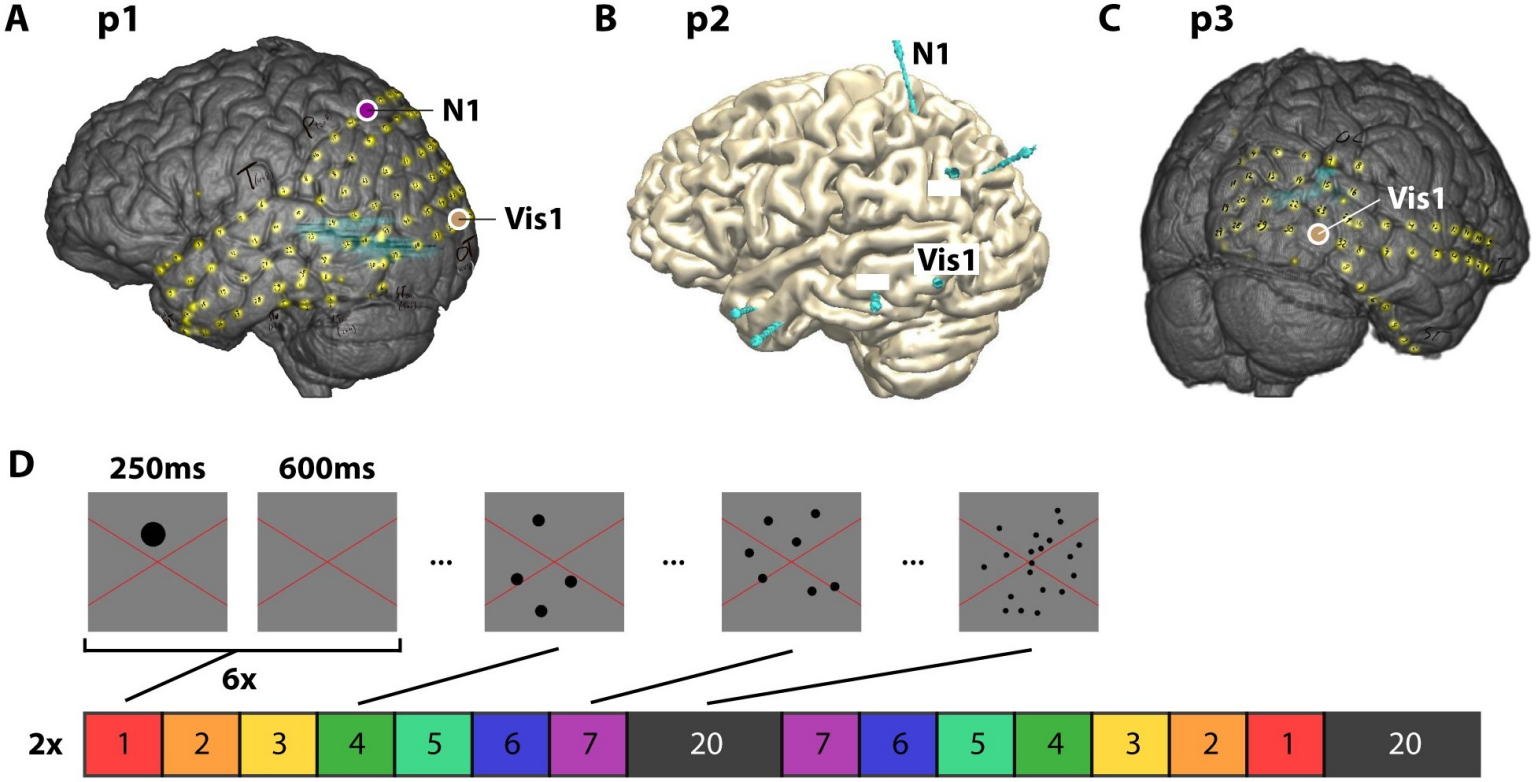
Electrode grid locations and stimulus sequence for the numerosity experiment. A) Lateral view with electrode locations (yellow dots) for participant 1. Purple circle indicates the location of parietal numerosity electrode 1 (N1). Brown circle indicates the location of occipital electrode 1 (Vis1). B) Lateral view with S-EEG needle locations (blue lines) for participant 2. Labelled needles were included in the analysis. N1 contained 1 numerosity-selective electrode (N1), and Vis1 contained multiple visual category-selective electrodes. C) Caudal-lateral view with electrode locations (yellow dots) for participant 3. Brown circle indicates the location of occipital electrode 1 (Vis1). D) Stimulus sequence and stimulus examples (cut-outs of the central portion of the display). Stimuli were presented for 250 ms, with a 600 ms (650–950 ms for participant 2) inter-stimulus interval. Stimuli were presented sequentially, with six (numerosity 1–7) or twelve (numerosity 20) consecutive presentations. The first presentation of each numerosity block were removed from analysis for these sequential numerosity runs. The coloured block sequence was repeated twice per run. Additional runs for participants 2 and 3 contained stimuli that were presented in a semi-randomized order.

Participant 1 was a 19-year old, left-handed female with Electrode grids covering parts of the left hemisphere (Fig 1A). Participant 2 was a 33-year old, right-handed female with seven S-EEG needles in the left hemisphere (Fig 1B). Participant 3 was a 19-year old, right-handed female with electrode grids covering parts of the right hemisphere (Fig 1C).

Two additional participants with surface electrode grids and four participants with S-EEG needles completed the experiment but were not included in further analyses because data contained excessive epileptic activity or electrodes did not cover regions-of-interest for this study due to differences between pre-operative planning and actual electrode location.

### Stimuli

Visual stimuli were presented to the participants on an NEC MultiSync E221N, 21.5-inch IPS monitor, with a resolution of 1920x1080 pixels, driven by an Acer Aspire E5-573G laptop. The screen was viewed at approximately 70 cm distance. Numerosity stimuli were presented using Presentation^®^ software (version 18.0, Neurobehavioral Systems, Inc., Berkeley, CA, www.neurobs.com). The stimulus code can be found at the Open Science Framework (https://osf.io/3w9sg/, DOI 10.17605/OSF.IO/3W9SG). Visual category stimuli were presented using MATLAB (2018, version R2018a. Natick, Massachusetts: The MathWorks Inc.) and Psychtoolbox-3 (https://psychtoolbox.org/). The code used for generating and presenting the visual category stimuli can be found at https://github.com/BAIRR01/BAIR_stimuli (see *stimMakeSpatiotemporalExperiment*.*m*) and at https://github.com/BAIRR01/vistadisp.

#### Numerosity stimuli

Numerosity stimuli consisted of one set of circles per display, randomly distributed within an invisible circle with a 50-pixel radius. Mean luminance gray background with a large diagonal red cross was presented at all times. This cross aided fixation and ran from one corner of the screen to the other. The number of circles per stimulus display included all integers from 1 to 7, and 20. Numerosity 20 was chosen because it is expected to be well outside the response range of neuronal populations responding most to small numerosities, while neurons responding to just contrast energy should respond most strongly to this energy-rich condition [28]. The total surface area of each set of circles was constant; hence the circles were larger when the stimulus display contained fewer of them. We chose to keep the total surface area constant to equate the amount of luminance. However, in previous studies we varied different visual features, where the dot cloud was unconstrained (all dots are the same sizes), dot cloud always occupied same total surface area, dot cloud always occupied same total edges, varied dot density and used random shapes other than dots. In all cases the same, or nearly the same, tuning to numerosity and the same numerosity maps were found [28,29,32]. On regular trials, the circles were black. In approximately 10% of the trials (two per numerosity on average), presented circles were white instead of black and participants had to respond to these trials by pressing a button. No numerosity-related judgments were required.

Stimuli were presented for 250 ms, followed by an inter-stimulus interval (ISI) of 600 ms during which only the mean luminance gray screen with the fixation cross was presented. Numerosities 1 to 7 were displayed sequentially in ascending order, then in descending order with six consecutive presentations per numerosity (Fig 1C). These ascending and descending sweeps were separated by twelve presentations of numerosity 20. The full sequence (ascending 1–7, then 20, descending 7–1, then 20) was repeated twice within each run. Each run started and ended with a 1200 ms mean luminance grey screen with a fixation cross, resulting in a total presentation time of 186 s per run. The stimulus sequence was near-identical to those used in recent numerosity-related fMRI paradigms [28,29]. For participants 2 and 3, additional runs were acquired in which numerosity stimuli were presented in a semi-randomized order with a variable ISI of 650–950 ms, and each numerosity was presented 24 times. The total presentation time was 225 s per run. Stimuli were otherwise identical to the sequential-order runs. The additional runs with a semi-randomized order were to evaluate whether the order of the stimuli may matter. We found similar results using the traditional systematic order and semi-randomized order, hence we combined conditions.

#### Visual category stimuli

To control for visual stimulation per se, we compared responses during our numerosity experiment with responses during an experiment in which grayscale, band-pass filtered (3 cycles per degree) images of faces, individual letters, or houses were presented on a mean luminance grey background. These stimuli were presented as part of a separate study that included a large variety of band-pass filtered pattern stimuli similar to those used in Kay and colleagues [33]. Each image measured 330 x 330 pixels and was presented in the centre of the screen with a small fixation cross overlaid; participants were instructed to fixate on the cross and press a button on a response pad every time the cross changed colour (from green to red or red to green). Fixation cross colour changes were created independently from the stimulus sequence and occurred at randomly chosen intervals ranging between 1000 and 5000 ms. Each presentation lasted 500 ms, with a variable ISI lasting 1250–1750 ms during which only the mean luminance grey screen and fixation cross were presented. The total number of presentations per run was 36 (twelve per stimulus category), with a total average run time of 52.9 s per run. There were two runs that each contained unique stimuli and uniquely randomized stimulus orders and ISIs, which were then held constant across participants and repeated within some participants (see below).

### Data acquisition

Data were acquired using a 128-channel recording system (Micromed, Treviso, Italy) with a 2048 Hz sampling rate and band-pass filtered between 0.15–536 Hz. Electrodes were localized using an MRI-co-registered computed tomography (CT) scan and projected to the cortical surface of each participant [34]. We collected one sequential-order numerosity and six visual category runs for participant 1. For participant 2, we collected six randomized-order numerosity runs, one sequential-order numerosity run, and six visual category runs. The sequential-order numerosity run did not yield any significant results, so all presented results for this participant are based on the randomized-order runs. For participant 3, we collected one sequential-order numerosity run, two randomized-order numerosity runs, and two visual category runs. The randomized-order runs showed similar results to the sequential-order run. All presented results are based on the sequential-order run, as this matches the stimulus configuration for participant 1. The number of runs collected per patient per experiment depended on the condition and availability of the patient.

Note that due to issues with trigger timing for numerosity recordings of participant 1, any time-related measurements should only be compared to electrodes within that same participant. The trigger timing was shifted by an unknown, but fixed delay of approximately 100 ms. Timepoint zero for participant 1 in the figures thus does not exactly coincide with stimulus onset, but represents a moment after stimulus onset that is the same for all epochs and electrodes. This is also the reason that averaging windows and baseline referencing for numerosity data from participant 1 deviate from participants 2 and 3.

### Data preprocessing

All data processing was performed using MATLAB (2018a, Mathworks, MA, USA) and the Fieldtrip toolbox [35]. Electrode signals were inspected by a clinical professional and excluded from the analysis if they showed major recording artifacts or excessive epileptic activity. Electrodes included in the analysis were not located over the clinically defined seizure focus. Electrodes on the parietal (participant 1 and 2), temporal (all participants), and occipital lobes (participant 1 and 3) were selected for analysis. For participant 2, one additional parietal electrode terminal was excluded from the analysis due to poor signal quality as a result of being located outside gray or white matter (electrode MPL1; S1 Fig). For participant 3, three additional electrodes were excluded from further analysis due to poor signal quality (electrodes Oc9, Oc12 and Oc18; S1 Fig). A total of 48 implanted electrodes were included for participant 1, and 53 electrodes for participant 3, based on cortical location and signal quality (see above; S1 Fig). A total of 21 electrode sites (terminals) on two S-EEG needles (one in the parietal and one in the occipito-temporal lobe) were selected for participant 2 (see above; S1 Fig). All time course data of each participant were first notch-filtered at 50 Hz and 2 octaves (100 and 150 Hz, filter width of 3 Hz) to remove line noise and subsequently re-referenced to the common average of all included electrodes.

### Numerosity experiment processing

All numerosity data were epoched from 500 ms before until 1000 ms after stimulus onset. For the sequential-order runs, the first presentation of each numerosity block was removed to exclude any numerosity change onset effects. For each run, this left 20 presentations per numerosity for numerosities 1–7, and 40 presentations of numerosity 20 for further analysis. For participant 2, this process was omitted for the randomized-order runs, resulting in 24 presentations per numerosity. We first removed the evoked signal (average over all epochs with the same numerosity) for each numerosity from the common average-corrected signals. Next, we calculated the power for each epoch using a fast Fourier transform (FFT) over a frequency range of 2–122 Hz in steps of 2 Hz with a sliding window width of 300 ms, tapered with a Hanning window to attenuate edge effects. The sliding time-window lay between 200 ms before and 700 ms after stimulus onset, in steps of 10 ms. The resulting power data were then normalized per frequency by dividing by the mean power for that frequency, so that the amplitudes at each frequency contributed equally to the resulting signal [36,37]. These normalized data for each presentation were subsequently baseline-corrected by subtracting the mean over 200 ms to 50 ms preceding stimulus onset (participants 2 and 3), or by subtracting the signal magnitude at 200 ms preceding trigger timing (participant 1). This difference between participants was dictated by an issue with trigger timing (see the section *Data Acquisition* for further details).

To identify electrodes of interest, average power spectral density (PSD) for each numerosity was plotted for each electrode. All further analyses were performed on the average normalized power over a high-frequency band of interest (HFB; 60–120 Hz) as the HFB has been shown to correlate with BOLD-fMRI [36,38,39]. Trials were excluded if their normalized power varied more than 0.5 before stimulus onset, or if the mean signal across 200–50 ms preceding the presumed stimulus onset varied by more than three standard deviations from the mean signal across numerosity presentations. Subsequently, tuning preferences were identified by taking the mean response for each numerosity over the HFB and a time window 0–250 ms (participant 1, see timing note in *Data Acquisition*) or 100–350 ms (participants 2 and 3) after stimulus onset.

For electrodes that responded to the numerosity stimuli we observed 3 qualitatively different response patterns: numerosity tuned, showing an increasing response with increasing numerosity (but without tuning for a specific numerosity), and a roughly equal response to all numerosity stimuli. In the main text we present, for each participant, detailed results for one example numerosity-tuned electrode (if present in that participant) and for one example untuned—but visually responsive—electrode (i.e. either showing an increasing or a similar response to increasing numerosity). Specifically, numerosity-tuned electrodes are presented for participants 1 and 2 (electrodes p1-N1 and p2-N1); Electrodes increasing their response with numerosity, but without tuning to a specific numerosity are presented for participants 1 and 2 (p1-Vis1 and p2-Vis1); and an electrode showing a similar response to all numerosity is presented for participant 3 (p3-Vis1). As shown in the Supplement (S2, S4 and S6 Figs) additional electrodes showing an increasing or a similar response to increasing numerosity were found. We chose not to report all these electrodes in the main text, because these untuned response patterns are not the main focus of the present study. In participants 1 and 3 there were no additional numerosity-tuned electrodes. In participant 2 there was one additional numerosity-tuned electrode (electrode MPL8, Supplementary S4 Fig). We do not report it in the main text, because it likely picked up neighbouring/overlapping cortical sources, as it was located on the same depth electrode next to the reported electrode p2-N1 and it was also tuned to numerosity 1. Responses for all analysed electrodes are presented in the supplementary materials (S2, S4 and S6 Figs). The responses to different numerosities were assessed by comparing the highest average numerosity response to the responses to other numerosities using a general linear model (GLM) analysis with each numerosity as a categorical variable.

### Control experiment processing

All processing steps for the visual categories data were identical to the numerosity data. However, exact parameters differed for some steps that are listed below.

Initial epochs were defined starting 200 ms before until 1250 ms after stimulus onset. Mean responses across time and frequency range of interest were taken from 100–350 ms after stimulus onset. Responses for visual categories and numerosity were then compared using a GLM analysis with the highest numerosity response as baseline, and each stimulus category as categorical variable. Responses for all electrodes are presented in the supplementary materials (S3, S5 and S7 Figs). Responses for the electrodes detailed above are also presented in the main text (Figs 3 and 4).

## Results

### High-frequency band power reveals numerosity tuned responses in parietal cortex

Numerosity-tuned electrodes that did not respond to visual category stimuli were found in participants 1 and 2. The numerosity-tuned electrodes in p1 and p2 covered a part of the parietal cortex that corresponds with the most robustly identifiable numerosity-selective topographic map in fMRI studies. In p3 there were no electrodes sampling this part of cortex.

**Fig 2:**
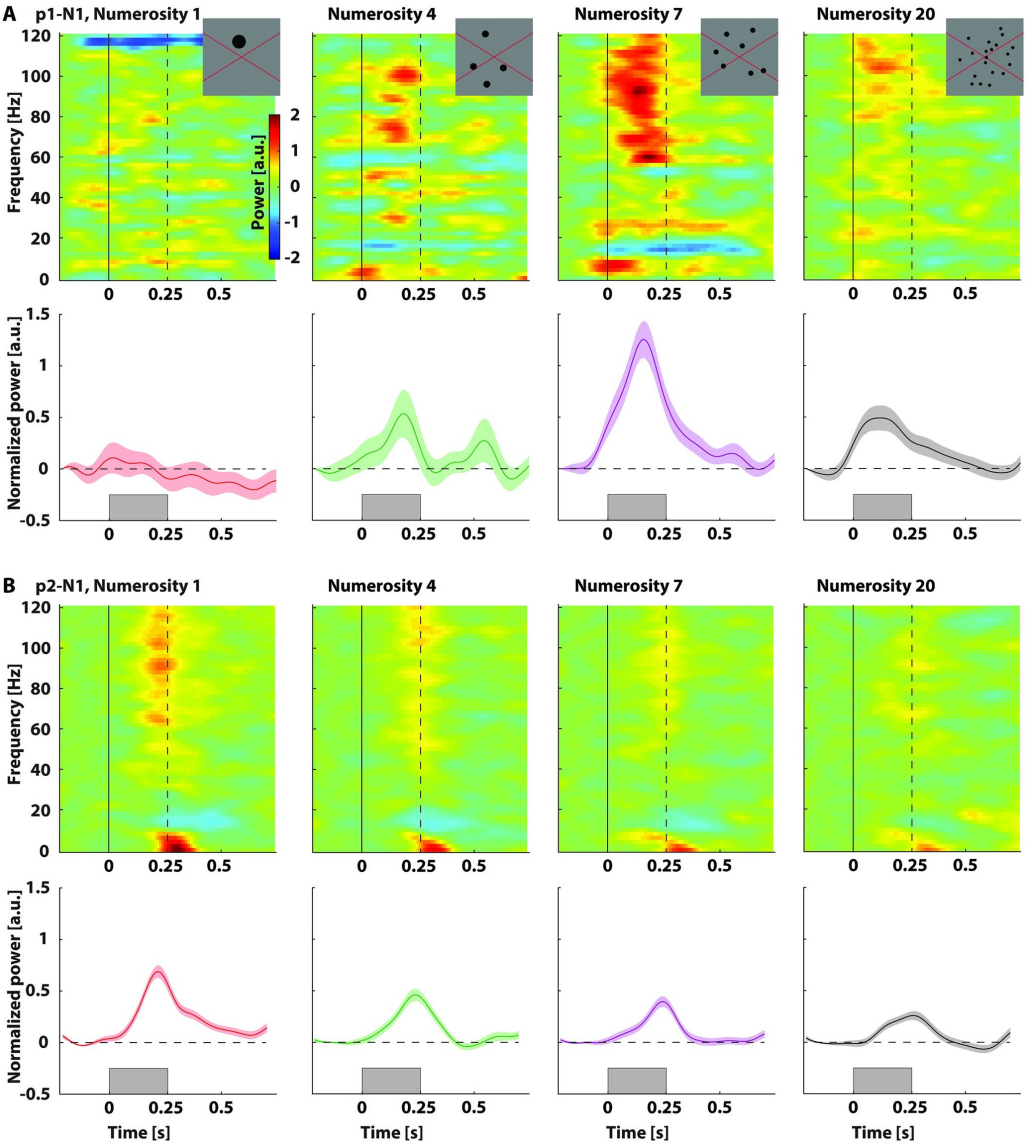
Responses to numerosity stimuli for participant 1(A) and 2(B). Top rows of each panel: Normalized and baseline-corrected power spectral density across frequency and time for the selected parietal electrode for numerosity 1, 4, 7, and 20 (example stimuli as insets). Solid black lines represent stimulus onset (see note on trigger timing of participant 1 in the Methods section), dashed black lines represent stimulus offset (total presentation time per epoch: 250 ms). Bottom rows of each panel: Normalized and baseline-corrected power in the high frequency band (60–120 Hz) over time. Shaded regions represent standard error of the mean across stimulus repetitions. Gray rectangles represent when a stimulus was presented. Normalization involved division by the mean power per frequency. Baseline correction involved subtraction of a pre-stimulus baseline interval.

**Fig 3:**
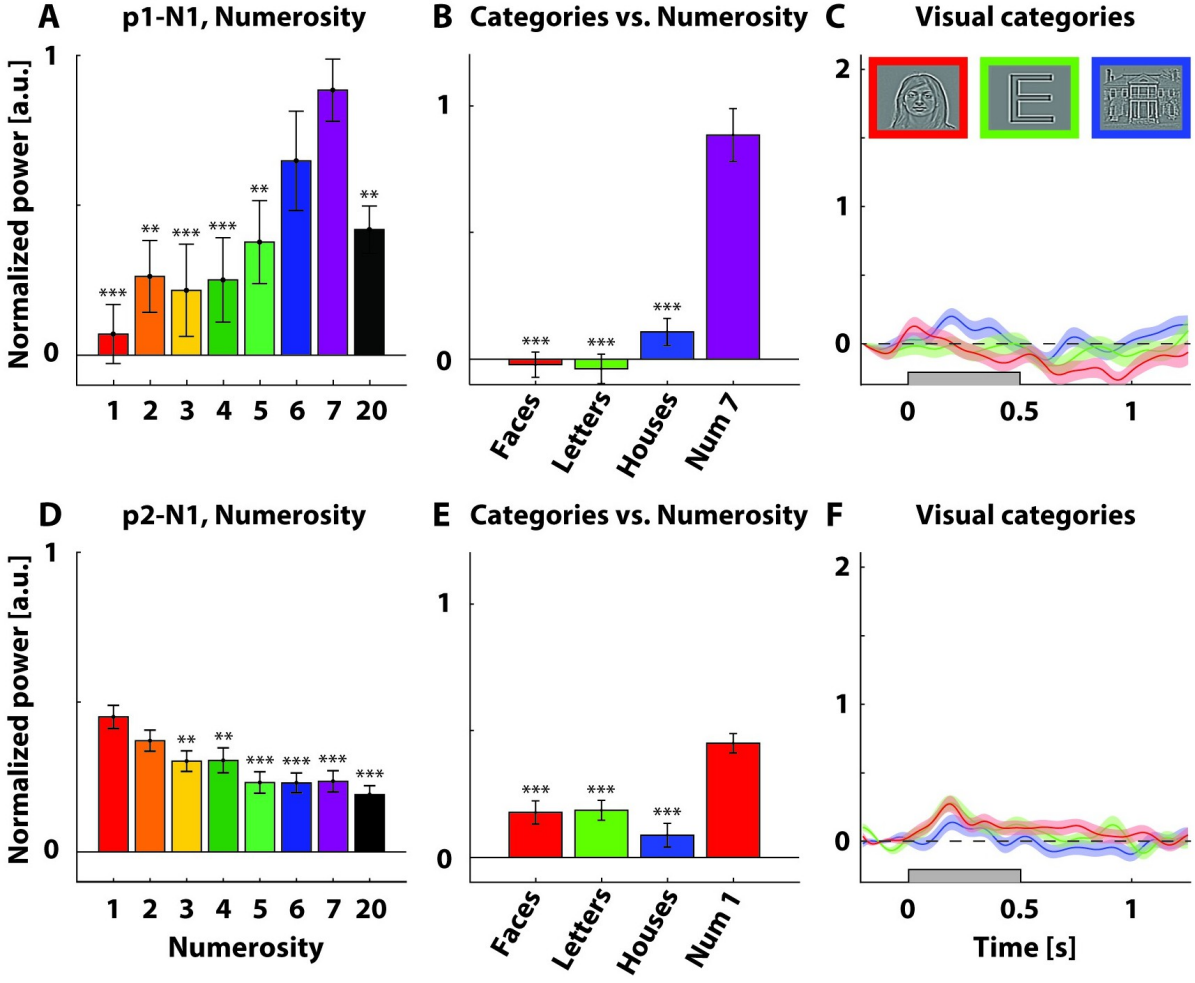
Selected parietal electrodes showed numerosity-specific responses. A, D) Mean normalized and baseline-corrected power across the high frequency band (HFB, 60–120 Hz) across a time window 0–250 ms (participant 1) and 100–350 ms (participant 2) after stimulus onset (see note on trigger timing of participant 1 in the Methods section) for selected numerosity-selective electrodes. B, E) Mean normalized and baseline-corrected power across HFB across 100–350 ms (both participants) after stimulus onset for visual category stimuli and mean normalized and baseline-corrected power as in 3A, D for numerosity 7 (B) and 1. Asterisks denote responses significantly different from the highest numerosity response. * = p<0.05, ** = p<0.01, *** = p<0.001. C, F) Mean normalized and baseline-corrected power across HFB for visual category stimuli. Gray rectangles represent stimulus presentation. Shaded regions represent standard error of the mean across stimulus repetitions. Inset (C): Stimulus examples for faces, letters, and houses categories. Normalization involved division by the mean power per frequency. Baseline correction involved subtraction of a pre-stimulus baseline interval.

Parietal electrode 1 in participant 1 (participant 1, numerosity-responsive electrode 1; p1-N1) PSD-plots showed a large response across the HFB to numerosity 7 and smaller responses to numerosities further away from 7 (Figs 2A and 3A). We performed a GLM analysis, showing that the responses of p1-N1 to numerosity 1, 2, 3, 4, 5, and 20 were significantly lower than for numerosity 7, *t(172)* = -4.36, -3.33, -3.58, -3.39, -2.72, -2.88, *p* < 0.001, 0.01, 0.001, 0.001, 0.01, 0.01 respectively. Responses to numerosity 6 were not significantly different from 7, *t(172)* = -1.26, *p* = 0.021. Responses to numerosity 7 were significantly higher than 0, *t(172)* = 6.70, *p* < 0.001 (Fig 3A). We thus observed tuning to numerosity 7 in p1-N1.

The PSD-plots of one electrode terminal in parietal S-EEG needle electrode N1 in participant 2 (p2-N1) showed a large response across the HFB to numerosity 1 and smaller responses to numerosities further away from that numerosity (Figs 2B and 3D). A GLM showed that responses to numerosity 3, 4, 5, 6, 7 and 20 were significantly lower than for numerosity 1, *t(980)* = -2.94, -2.87, -4.39, -4.37, -4.30, -5.11, *p* < 0.01, 0.01, 0.001, 0.001, 0.001, 0.001 respectively. Responses to numerosity 1 were significantly higher than 0, *t(980)* = 12.77, *p* < 0.001. Responses to numerosity 2 were not significantly different from 1, *t(980)* = -1.59, *p* = 0.11.

Next, we compared the numerosity responses to the responses to visual category stimuli (Fig 3B, 3C, 3E and 3F). In p1-N1, responses to faces, letters, and houses were significantly lower than responses to 7, *t(223)* = -7.83, *p* < 0.001, *t(223)* = -8.03, *p* < 0.001, and *t(223)* = -6.52, *p* < 0.001 respectively (Fig 3B and 3C). Likewise, in p2-N1 responses to face, letter, or house-stimuli were significantly lower than those related to numerosity 1, *t(330)* = -4.63, *p* < 0.001, *t(330)* = -4.20, *p* < 0.001, and *t(330)* = -6.20, *p* < 0.001 respectively (Fig 3E and 3F).

Thus, we observed a clear tuning to numerosity 7 in p1-N1 and a clear tuning to numerosity 1 in p2-N1, with decreased responses further away from the preferred numerosity. Moreover, both electrodes responded selectively to numerosity and significantly less to faces, letters and houses.

### Response patterns in occipital and temporal cortex do not show numerosity tuning

Response patterns in occipital and temporal cortex either showed increasing responses with increasing numerosity, or responded to all numerosities similarly. To illustrate this, we show the results for one occipital electrode for each participant below. All analysed occipital and temporal electrodes with responses to numerosity stimuli are shown in the supplement.

**Fig 4:**
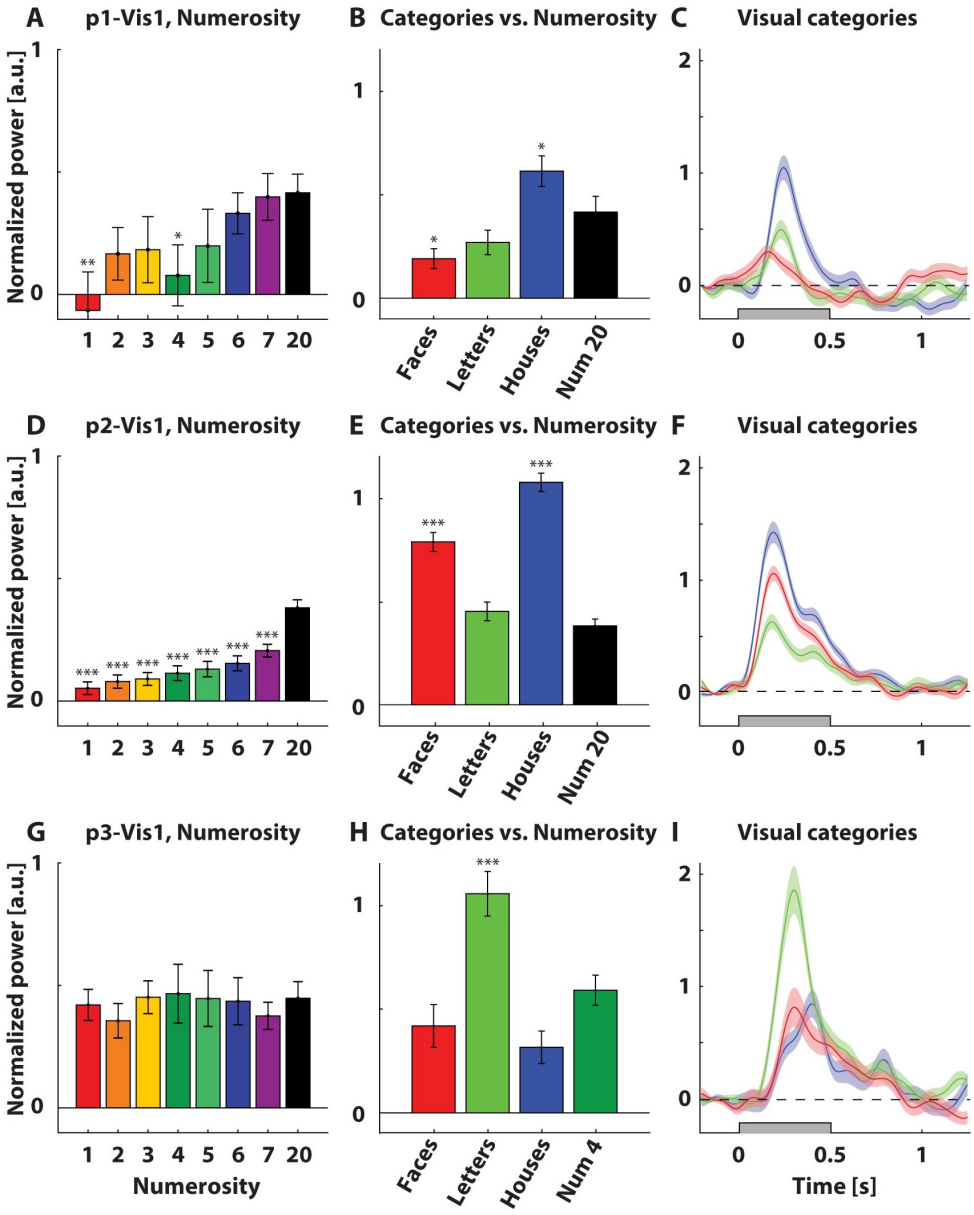
Occipital and temporal electrodes showed no numerosity-specific responses. A, D, G) Mean normalized and baseline-corrected power across the high frequency band (HFB, 60–120 Hz) across a time window 0–250 ms (participant 1) and 100–350 ms (participant 2, 3) after stimulus onset for visually responsive electrodes. B, E, H) Mean normalized and baseline-corrected power across HFB across 100–350 ms (all participants) after stimulus onset for visual category stimuli and mean normalized and baseline-corrected power as in 3A, D, G for numerosity 2 (B), 20 (E), and 4 (H). Asterisks denote responses significantly different from the highest numerosity response. * = p<0.05, ** = p<0.01, *** = p<0.001. C, F, I) Mean normalized and baseline-corrected power across HFB for visual category stimuli. Gray rectangles represent when a stimulus was presented. Shaded regions represent standard error of the mean across stimulus repetitions. Normalization involved division by the mean power per frequency. Baseline correction involved subtraction of a pre-stimulus baseline interval.

Occipital electrode p1-Vis1 showed no specific numerosity-tuned responses, but rather a general response to most numerosities (Fig 4A). Responses to numerosity 20 were significantly higher than 0, *t(172)* = 4.75, *p* < 0.001. Responses to numerosity 4 were significantly lower than to numerosity 20, *t(172)* = -2.22, *p* = 0.03. Responses to other numerosities did not differ from numerosity 20, *t(172)* = -1.64–0.11, *p* = 0.10–0.91. Responses to faces were significantly lower than responses to numerosity 20, *t(230)* = -2.27, *p* = 0.02. Responses to houses were significantly higher than responses to numerosity 20, *t(230)* = 1.98, *p* = 0.05 respectively, while responses to letters were not significantly different, with *t(230)* = -1.72, *p* = 0.09 (Fig 4B and 4C).

Occipital electrode p2-Vis1 showed no specific numerosity-tuned responses, but rather an increasing response with increasing numerosity (stimulus energy, Fig 4D). Responses to numerosity 20 were significantly higher than 0, *t(1002)* = 13.26, *p* < 0.001. Responses to lower numerosities were significantly lower than responses to numerosity 20, with subsequent numerosities showing subsequently smaller responses, *t(1002)* = -4.27–8.10, all *p* < 0.001. Responses to faces and houses were significantly higher than responses to numerosity 20, with *t(332)* = 7.19, *p* < 0.001 and *t(332)* = 12.34, *p* < 0.001, while responses to letters were not significantly different, *t(332)* = 1.25, *p* = 0.21 (Fig 4E and 4F).

Occipital electrode p3-Vis1 showed no specific numerosity-tuned responses, but rather a general response to all numerosities (Fig 4G). Responses to numerosity 4 were significantly higher than 0, *t(149)* = 4.76, *p* < 0.001. Responses to other numerosities were not significantly different from responses to numerosity 4, *t(149)* = -0.87–0.11, *p* = 0.39–0.91. Responses to letters were significantly higher than responses to numerosity 4, *t(80)* = 3.64, *p* < 0.001, while responses to faces and houses were not significantly different, with *t(80)* = -0.29, *p* = 0.78, and *t(80)* = -0.93, *p* = 0.36 respectively (Fig 4H and 4I).

## Discussion

We describe intracranial recordings of numerosity-tuned neuronal populations in human parietal cortex (responses were tuned to 7 and 1 respectively). These electrodes did not respond to images of faces, letters, and houses. In occipital cortex, we found either a relatively flat, or an increasing response with numerosity but no tuning to a specific numerosity. In contrast to numerosity-tuned electrodes, these occipital electrodes showed large responses to images of faces, letters, or houses. Thus, we show that the numerosity-tuned populations are distinct from the response pattern of electrodes that respond to visual information per se. These human intracranial recordings bridge the gap between the animal-model electrophysiological recordings and human BOLD fMRI measurements of numerosity tuned neural populations organized in topographic maps in human parietal cortex.

An increasing response with increasing numerosity, was observed for some occipital sites (Fig 4D), likely reflecting responses of neuronal populations sensitive to stimulus energy, as increasing numerosity of a stimulus display increases the contrast energy. This type of response is typically found in the visual system [32]. Responses of other occipital/temporal sites with a flat tuning were consistent with neuronal populations that responded non-selectively to visual information.

The locations of the numerosity-tuned electrodes are consistent with numerosity responses in parietal cortex as identified in previous fMRI studies [7,23,25,28]. As the numerosity-tuned electrodes showed different response preferences (numerosity 7 for participant 1, and numerosity 1 for participant 2), these findings are compatible with the presence of numerosity maps in humans, with neuronal populations tuned to different numerosities [27–30]. The chances of finding tuning specifically to numerosity 1 and 7 may be larger than other numerosities, considering that numerosity 1 spans a relatively large portion of the numerosity map found in fMRI studies, and populations tuned to 7 may include neurons tuned to numerosities larger than 7, but smaller than 20. Although these findings by themselves do not prove the existence of a topographic numerosity map, they are in line with such topographic organization.

Intracranial recordings measure a different aspect of neuronal activity when compared to fMRI, with intracranial recordings measuring neuronal population activity and fMRI measuring the hemodynamic consequences of neuronal activity. Even though these fMRI signals are correlated with HFB power [36,38–40], the relationship between the two is not completely clear. Here, we contribute to converging evidence of numerosity tuned neurons in humans.

### Limitations

As is common for intracranial recordings, we did not have experimental control over electrode placements, hence we were not able to ‘search’ for numerosity tuned populations but rather had to rely on the clinically informed coverage happening to cover them. This likely explains the absence of numerosity-selective electrodes in participant 3, as no parietal areas were covered in this participant.

As the numerosity stimuli in the sequential-order runs were presented in a predictable order, order effects might have been present in the data. To reduce these effects, we removed every first presentation from a stimulus block with the same numerosity. We also compared ascending and descending sequences within these runs and found similar tuning in both. For the randomized-order runs any order effects are assumed to be averaged out.

## Conclusion

We provide intracranial recordings of numerosity-tuned neuronal populations in human parietal cortex, thereby further linking animal electrophysiology and fMRI findings on numerosity tuning. We demonstrate parietal neural populations with tuning to numerosities 1 and 7 in a high frequency band (60–120 Hz) that has been shown to be correlated with fMRI signals.

## Supporting information

S1 Fig Electrode grid locations of analysed electrodes. A) Lateral view with electrode locations (yellow dots) for participant 1. B) Lateral view with S-EEG needle locations (blue lines) for participant 2. C) Caudal-lateral and medial view with electrode locations (yellow dots) for participant 3. Electrode sites labelled N1 (purple) or Vis1 (brown) are detailed in the main text.
(TIF)

S2 Fig Mean normalized and baseline-corrected power (60-120Hz; 0-250ms after stimulus onset trigger) in response to all tested numerosities for all processed electrodes of participant 1. Asterisks denote responses significantly different from the highest numerosity response. * = p<0.05, ** = p<0.01, *** = p<0.001.
(TIF)

S3 Fig Mean normalized and baseline-corrected power (60-120Hz; 100-350ms after stimulus onset) in response to visual category stimuli for all processed electrodes of participant 1. F = faces; L = letters; H = houses.
(TIF)

S4 Fig Mean normalized and baseline-corrected power (60-120Hz; 100-350ms after stimulus onset) in response to all tested numerosities for all processed electrodes of participant 2. Asterisks denote responses significantly different from the highest numerosity response. * = p<0.05, ** = p<0.01, *** = p<0.001.
(TIF)

S5 Fig Mean normalized and baseline-corrected power (60-120Hz; 100-350ms after stimulus onset) in response to visual category stimuli for all processed electrodes of participant 2. F = faces; L = letters; H = houses.
(TIF)

S6 Fig Mean normalized and baseline-corrected power (60-120Hz; 100-350ms after stimulus onset) in response to all tested numerosities for all processed electrodes of participant 3. Asterisks denote responses significantly different from the highest numerosity response. * = p<0.05, ** = p<0.01, *** = p<0.001.
(TIF)

S7 Fig Mean normalized and baseline-corrected power (60-120Hz; 100-350ms after stimulus onset) in response to visual category stimuli for all processed electrodes of participant 3. F = faces; L = letters; H = houses.
(TIF)
