# Supplementary figures and images for: Intracranial recordings show evidence of numerosity tuning in human parietal cortex
Source: PLoS One. 2022 Aug 3;17(8):e0272087. doi: 10.1371/journal.pone.0272087 (PMC9348694; doi:10.1371/journal.pone.0272087)

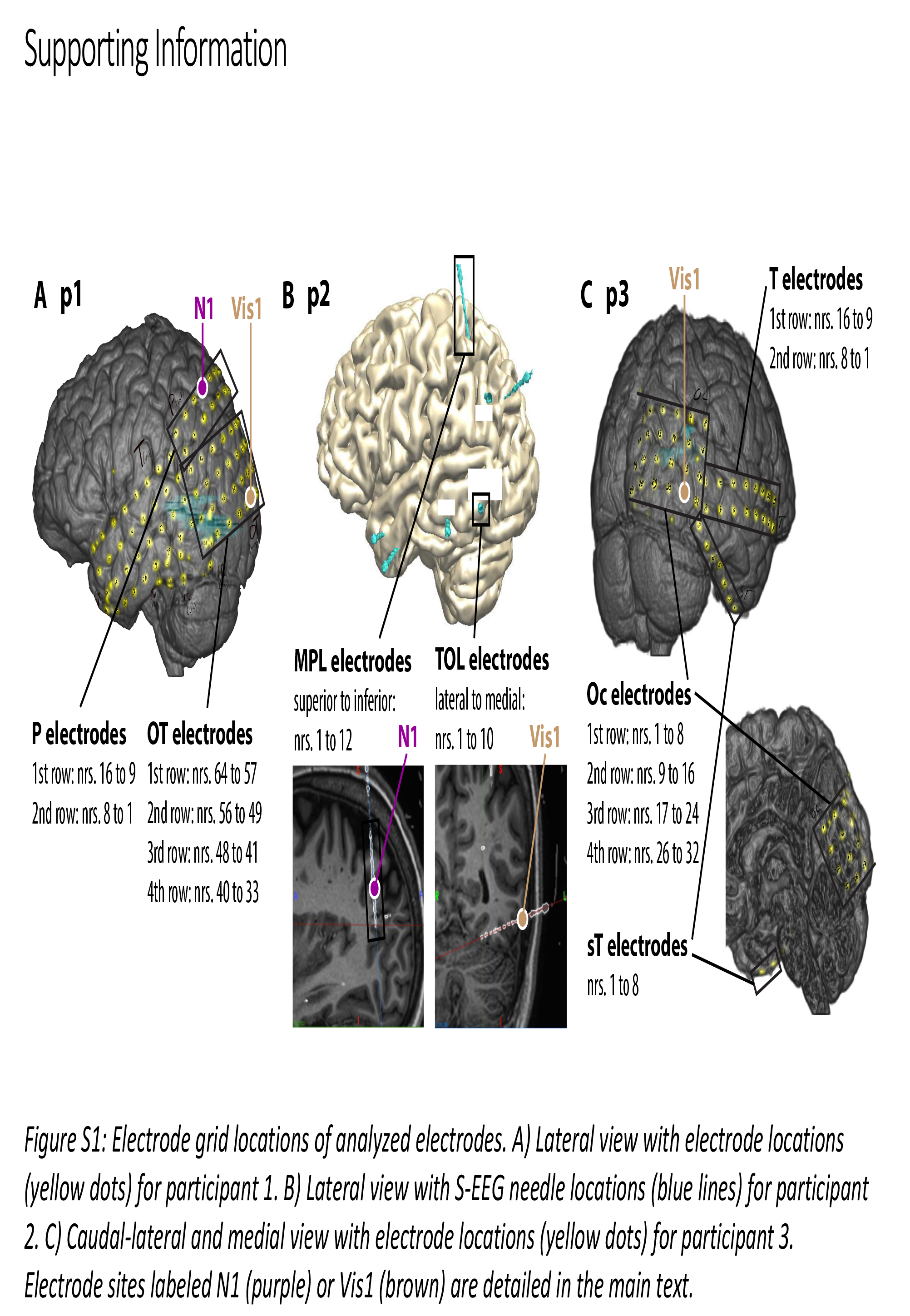

Supplement: S1 Fig — A) Lateral view with electrode locations (yellow dots) for participant 1. B) Lateral view with S-EEG needle locations (blue lines) for participant 2. C) Caudal-lateral and medial view with electrode locations (yellow dots) for participant 3. Electrode sites labelled N1 (purple) or Vis1 (brown) are detailed in the main text. (TIF) [file pone.0272087.s001.tif]

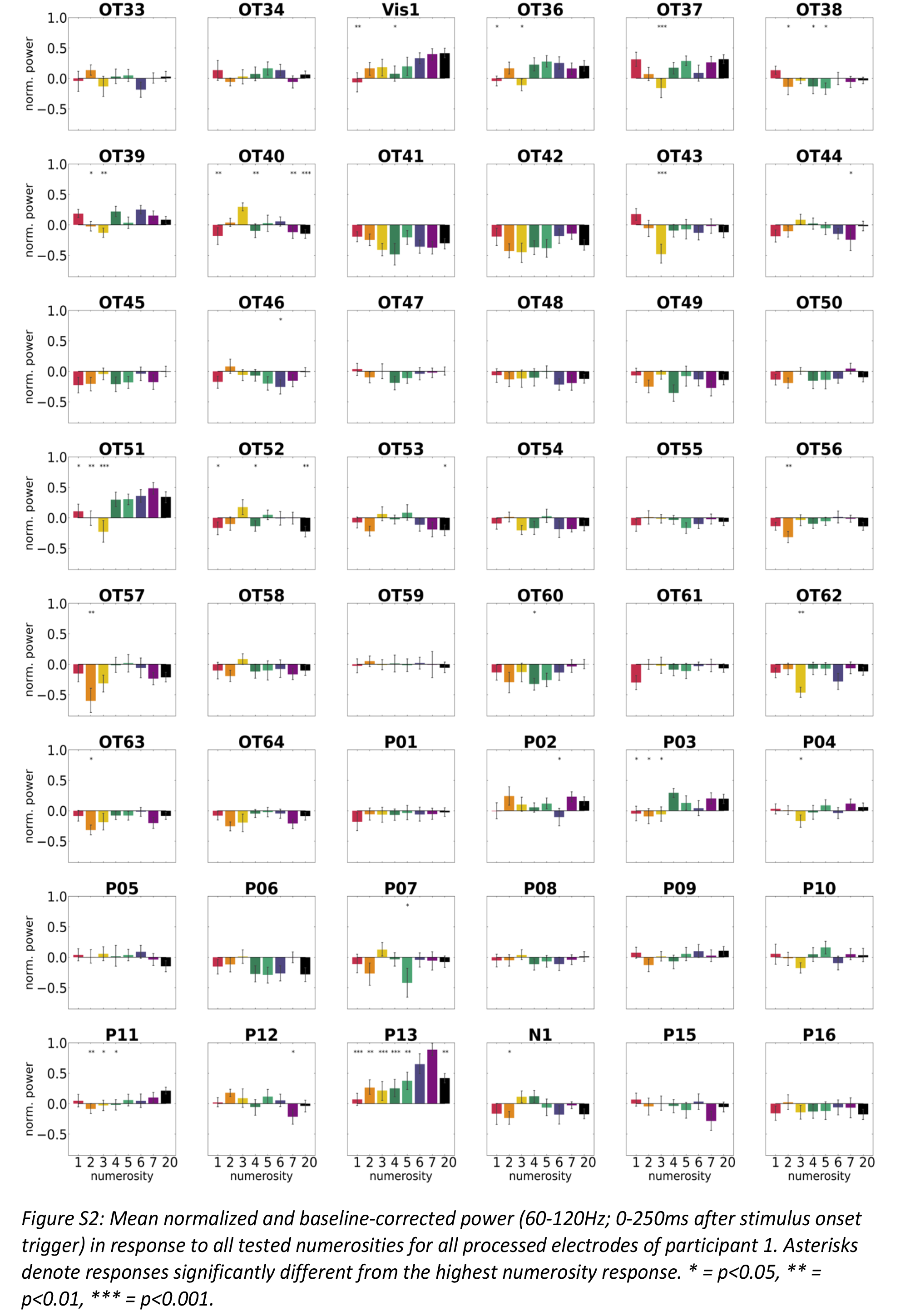

Supplement: S2 Fig — Asterisks denote responses significantly different from the highest numerosity response. * = p<0.05, ** = p<0.01, *** = p<0.001. (TIF) [file pone.0272087.s002.tif]

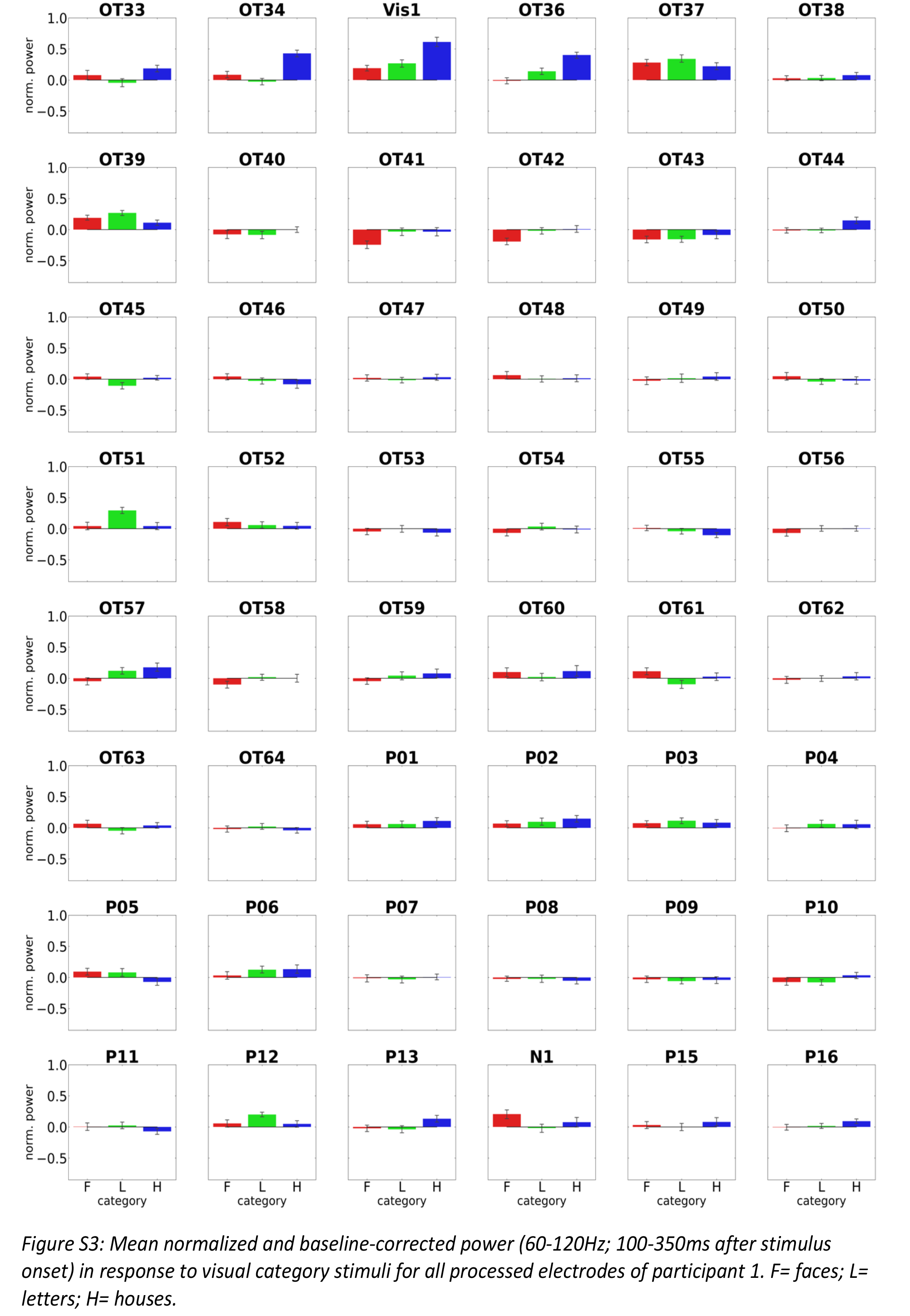

Supplement: S3 Fig — F = faces; L = letters; H = houses. (TIF) [file pone.0272087.s003.tif]

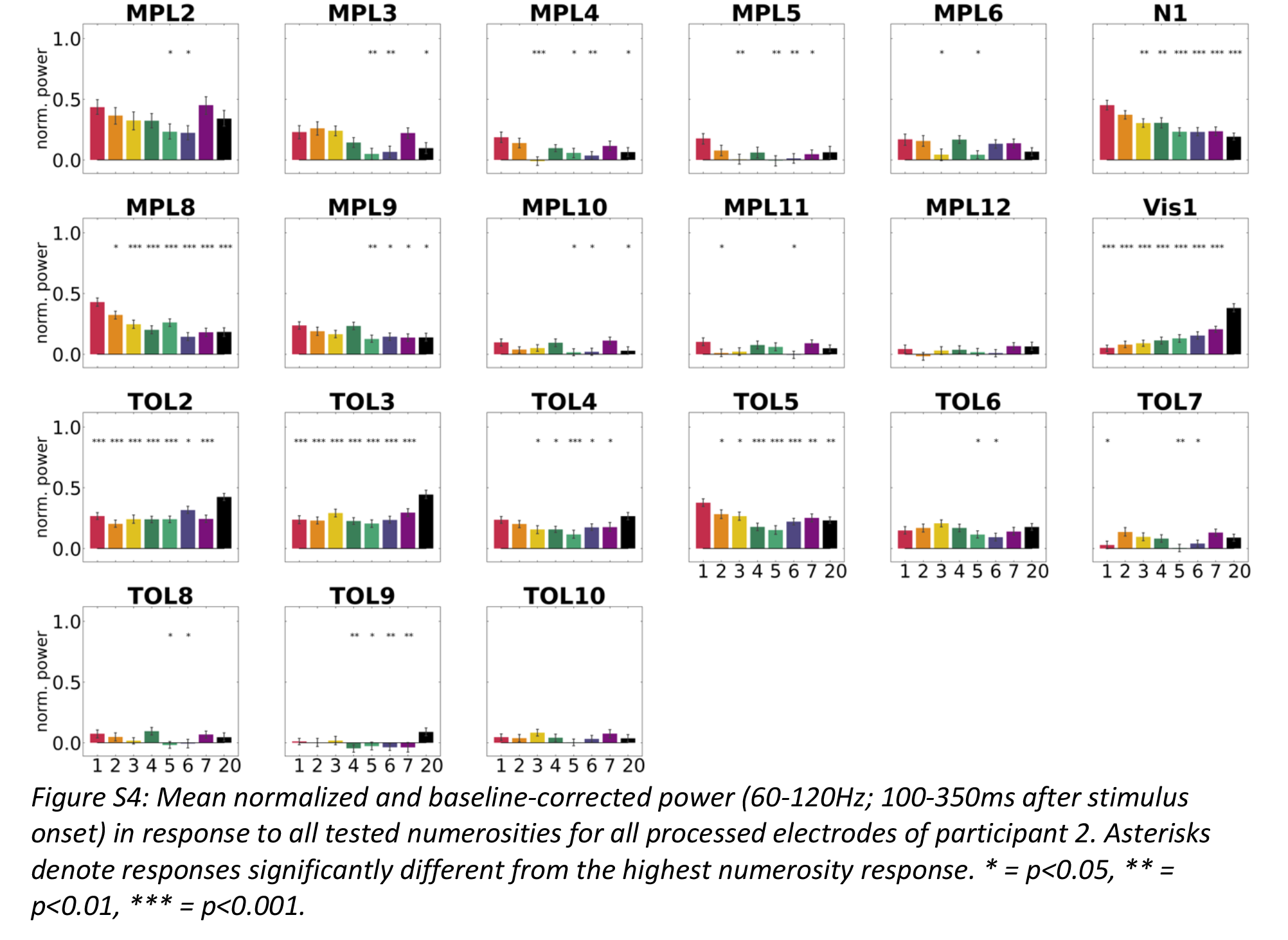

Supplement: S4 Fig — Asterisks denote responses significantly different from the highest numerosity response. * = p<0.05, ** = p<0.01, *** = p<0.001. (TIF) [file pone.0272087.s004.tif]

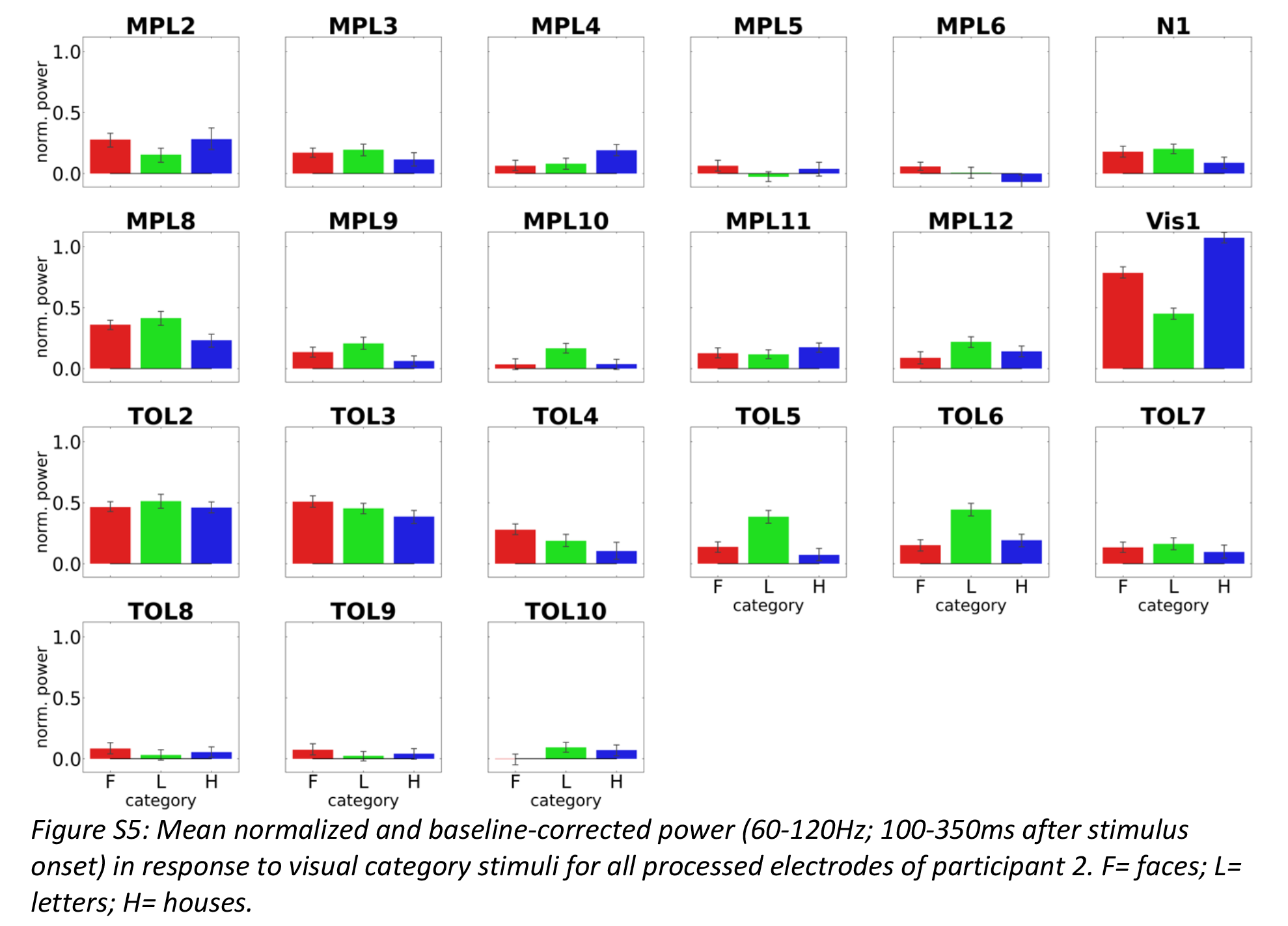

Supplement: S5 Fig — F = faces; L = letters; H = houses. (TIF) [file pone.0272087.s005.tif]

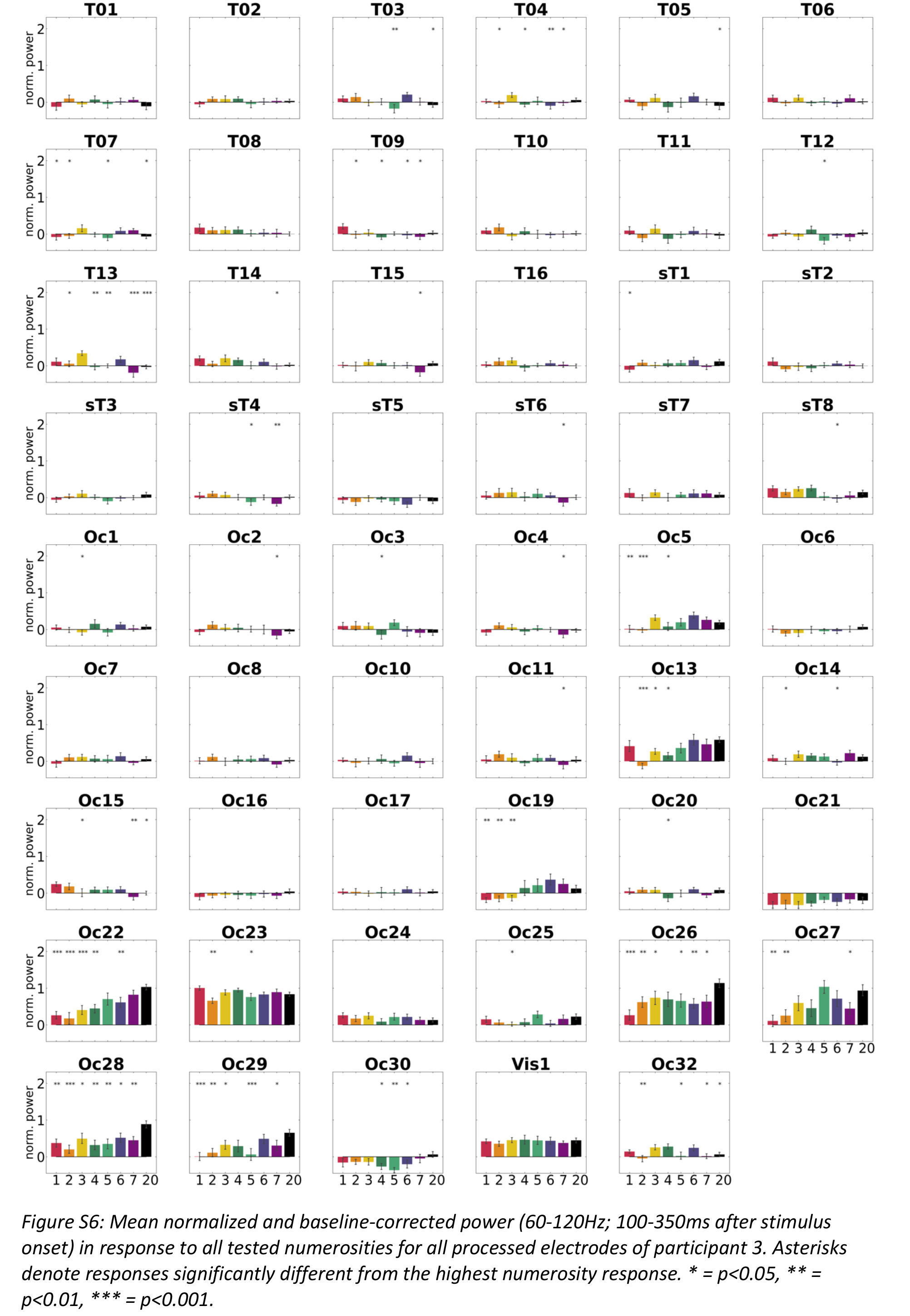

Supplement: S6 Fig — Asterisks denote responses significantly different from the highest numerosity response. * = p<0.05, ** = p<0.01, *** = p<0.001. (TIF) [file pone.0272087.s006.tif]

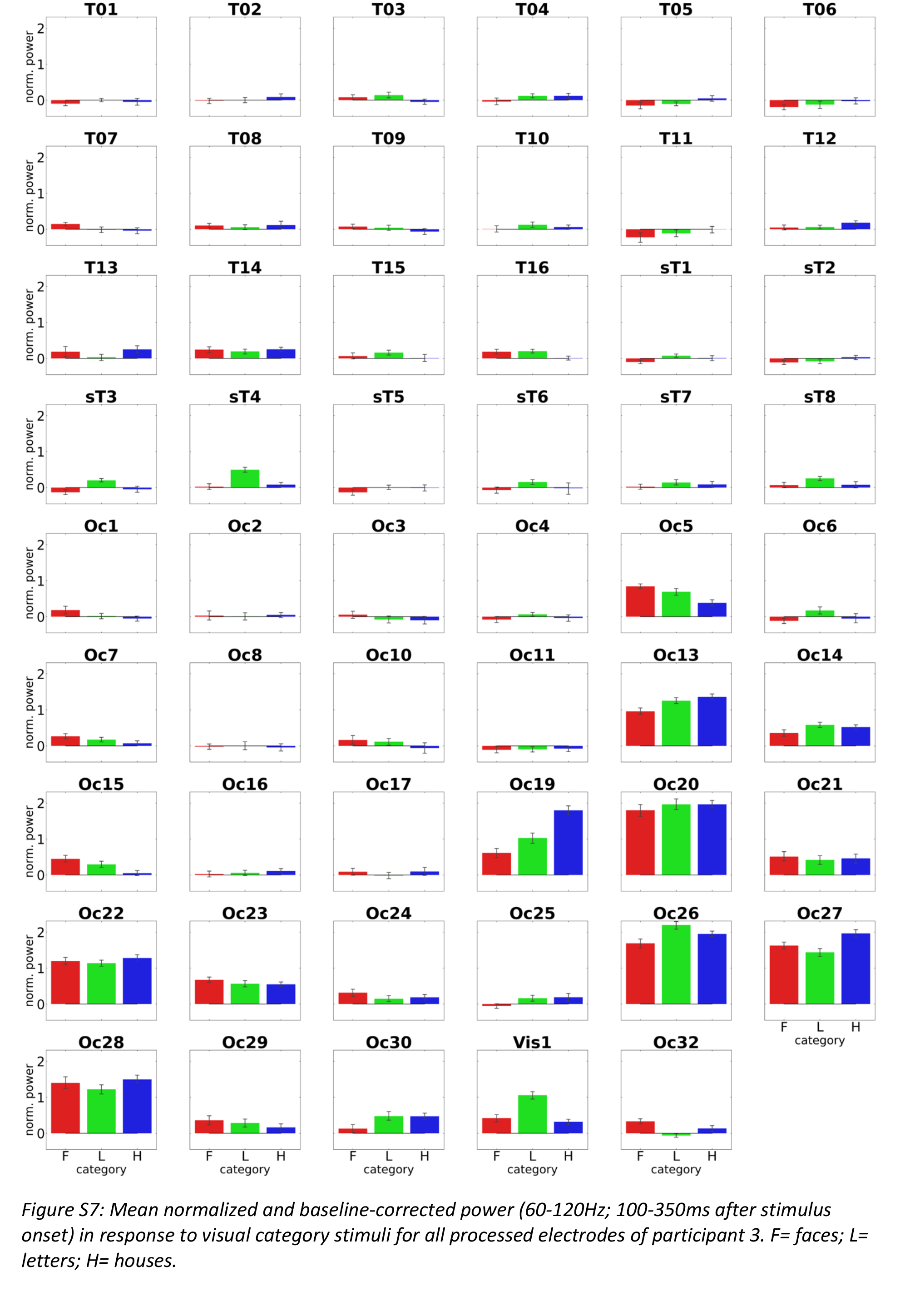

Supplement: S7 Fig — F = faces; L = letters; H = houses. (TIF) [file pone.0272087.s007.tif]
